# Temporal transcriptional control of neural induction in human induced pluripotent stem cells

**DOI:** 10.3389/fnmol.2023.1139287

**Published:** 2023-05-05

**Authors:** Shakti Gupta, Lucia Dutan Polit, Michael Fitzgerald, Helen A. Rowland, Divya Murali, Noel J. Buckley, Shankar Subramaniam

**Affiliations:** ^1^Department of Bioengineering, University of California, San Diego, San Diego, CA, United States; ^2^Maurice Wohl Clinical Neuroscience Institute, King’s College London, London, United Kingdom; ^3^Department of Psychiatry and Kavli Institute for Nanoscience Discovery, University of Oxford, Oxford, United Kingdom; ^4^Departments of Computer Science and Engineering, and Cellular and Molecular Medicine, University of California, San Diego, San Diego, CA, United States

**Keywords:** pluripotency, induced pluripotent stem cell, neural induction, transcription, OTX2

## Abstract

**Introduction:**

Neural induction of human induced pluripotent stem cells represents a critical switch in cell state during which pluripotency is lost and commitment to a neural lineage is initiated. Although many of the key transcription factors involved in neural induction are known, we know little of the temporal and causal relationships that are required for this state transition.

**Methods:**

Here, we have carried out a longitudinal analysis of the transcriptome of human iPSCs undergoing neural induction. Using the temporal relationships between the changing profile of key transcription factors and subsequent changes in their target gene expression profiles, we have identified distinct functional modules operative throughout neural induction.

**Results:**

In addition to modules that govern loss of pluripotency and gain of neural ectoderm identity, we discover other modules governing cell cycle and metabolism. Strikingly, some of these functional modules are retained throughout neural induction, even though the gene membership of the module changes. Systems analysis identifies other modules associated with cell fate commitment, genome integrity, stress response and lineage specification. We then focussed on OTX2, one of the most precociously activated transcription factors during neural induction. Our temporal analysis of OTX2 target gene expression identified several OTX2 regulated gene modules representing protein remodelling, RNA splicing and RNA processing. Further CRISPRi inhibition of OTX2 prior to neural induction promotes an accelerated loss of pluripotency and a precocious and aberrant neural induction disrupting some of the previously identified modules.

**Discussion:**

We infer that OTX2 has a diverse role during neural induction and regulates many of the biological processes that are required for loss of pluripotency and gain of neural identity. This dynamical analysis of transcriptional changes provides a unique perspective of the widespread remodelling of the cell machinery that occurs during neural induction of human iPSCs.

## 1. Introduction

Human iPSCs serve as excellent cellular models for neural lineage specification. Neuronal development from iPSC offers the potential to discover molecular pathways governing neurological pathologies and provide screens for target and therapeutic discovery. Furthermore, recent studies have demonstrated that iPSC-derived neurons reflect key aspects of clinical phenotype of the individual from whom the iPSCs were derived in a patient-specific manner (Lagomarsino et al., 2021; Ng et al., 2022), opening the door to using iPSC-derived neurons to discover pathogenic mechanisms and deliver personalized treatment. However, realization of this vision is hampered by the considerable variation that exists in the efficiency with which individual iPSCs generate neurons (Volpato and Webber, 2020). Differentiating iPSCs to neurons is a multi-step process and each step introduces more potential for variation. The first step on this developmental journey is neural induction, the initiating process by which pluripotent iPSCs become committed to a neural fate and transition to multipotent neural precursors (Stern, 2006). Understanding the temporally-relevant molecular mechanisms that drive this transition is critical to driving consistent differentiation toward neurons.

Our current understanding of vertebrate neural induction is based on the ‘default model’ whereby the neural plate emerges from dorsal ectoderm *via* blockade of BMP/NODAL signaling through release of inhibitors and other inductive and permissive signals originating from the organizer and flanking epidermis (Hemmati-Brivanlou and Melton, 1997). This default model of neural induction has now been shown to be broadly conserved across mammals and has also been demonstrated in human embryonic stem cells (Ozair et al., 2013). Neural induction can be recapitulated in human pluripotent stem cells using dual SMAD inhibition to block BMP/NODAL signaling (Chambers et al., 2009), a process that is sufficient to initiate neural induction and concurrently suppress pluripotency. Several key transcription factors expressed early during neural induction have been identified including some, such as PAX6 and ZNF521, that are necessary and sufficient to drive neural induction in human ESCs (Zhang et al., 2010; Kamiya et al., 2011). Others such as ZEB2 and NR2F2 have a dual function and act to concurrently directly suppress pluripotency factors such as POUF51 and activate pro-neural induction factors such as PAX6 (Ozair et al., 2013). Changes in cell state during development require wholesale remodeling of the cells’ regulatory and physical landscape reflected in timely and coordinated regulation of the epigenome, proteome and metabolome. The regulatory factors that orchestrate this transition from pluripotency to a specified lineage act transiently over specific time periods to achieve intermediate end point cell states. For the most part the resultant regulatory networks have been inferred from transcriptome data harvested from the end points of the developmental trajectory and the resulting interaction maps are static and fail to capture the dynamical aspects of the network throughout the developmental transition. However, deciphering the temporal aspects of involvement of specific regulatory factors is now possible from studying the transcription landscape changes during the lineage specification and it is this perspective that motivated the work presented here.

In this study, our goal was primarily to investigate the early-stage cell state regulators during neural induction using a fine-grained transcriptomic analyses. We harvested transcriptome data throughout 8 days of neural induction of human iPSCs, and captured the temporal profile of key transcription factors and their target genes to identify functional modules that are operative during neural induction. Our systems analysis revealed, in addition to canonical modules associated with loss of pluripotency and gain of neural identity, a number of additional modules, including those that regulate cell cycle, metabolism, genome integrity and stress. Two key factors include KEAP and NRF2, genes that regulate cellular response to numerous stressors, including oxidative, metabolic stressors (Baird and Yamamoto, 2020). Strikingly, even though some of these modules are operative throughout neural induction, the gene membership of the modules changes, indicating a degree of redundancy of gene membership in retaining module functionality. In order to validate the power of our analysis, we used CRISPRi to knock down expression of one of the most precociously expressed transcription factors during neural induction, OTX2 (Acampora et al., 2013). We find that loss of OTX2 leads to an accelerated loss of pluripotency and an accelerated and aberrant neural induction and disrupts many of the previously identified modules. Our analysis of the dynamics of transcriptional changes provides a unique perspective of the dynamics of transcriptional modules and widespread remodeling of the cell machinery, illustrating the tight coupling between changes in cell state and lineage and regulation of basic cellular machinery that occurs during neural induction of human iPSCs.

## 2. Materials and methods

### 2.1. Neural differentiation from human ESCs and iPSCs

Three human induced pluripotent stem cell (hiPSC) lines (CTR M1 04, CTR_M2_42 and CTR_M3_36s), derived from neurotypic males from ages ranging between 35 to 55 years, were used to generate two time series namely 0–48 h (h) and 0–8 days (d). These iPSC lines were maintained in Essential 8™ medium (Thermo Fisher) without antibiotics at 37°C, 5% CO_2_, 5% O_2_ on Geltrex™ (Thermo Fisher) coated plates. Differentiation of iPSCs was carried out as previously described (Shi et al., 2012; Lee et al., 2020). Briefly. As iPSCs reached 100% confluence, media was switched from Essential 8™ medium (Thermo Fisher) to neural induction medium. Neural induction medium comprised 50% Neurobasal medium (Thermo), including B-27 (Thermo) and GlutaMAX (Thermo), and 50% DMEM/F12 (Thermo) supplemented with N-2 (Thermo) and GlutaMAX (Thermo). The dual SMAD inhibitors (2i) 100 nM LDN193189 (Sigma Aldrich) and 10 μM SB431542 (Sigma Aldrich) with and without the inclusion of the WNT inhibitor XAV939 were also added for the first 7 days. The medium was changed daily throughout the differentiation. Data generated by all three cell lines and two neural induction protocols showed the similarity based on the principle component analysis (PCA) and changes in key pluripotency and neuroectoderm transcription factors (Supplementary Figure S1). Thus, The CTR M3 36S line was analyzed for the 0–48 h time series containing 8 time points, 0, 2, 4, 8, 18, 24, 30, and 48 h and the CTR M1 04 line was analyzed for 0–8 days time series containing 7 time points, 0, 1, 2, 3, 4, 6, and 8 d. The cell line CTR M2 42 was analyzed at time points 0,1,2,3,4,6 and 8 and was used exclusively for the generation of the PCA.

### 2.2. RNA isolation, analysis, library preparation, and sequencing

RNA was extracted at time points: day 0 (before the addition of differentiation media) and days 1, 2, 3, 4, 6 and 8 after induction. RNA was harvested and lysed with Trizol reagent (Life technologies, 15,596,026) and isolated by centrifugation with 100% Chloroform, following by 100% isopropanol and lastly by 75% ethanol. The RNA was purified with Qiaquick PCR purification kit (Quiagen, 28,106) and quantified with the NanoDrop 1,000 Spectrophotometer (Thermo scientific). The quality control was assessed by analyzing the RNA with the Agilent RNA 6000 nano Kit (Agilent technologies, 5,067–1,511) in combination with the Agilent 2,100 Bioanalyzer system.

RNAseq libraries were prepared with the Truseq RNA Kit. Briefly, mRNA was purified from total RNA followed by cDNA synthesis. Subsequently, the cDNA was end-paired, A-tailed and custom indexing adapters were ligated. Samples were size selected and pooled for sequencing. Libraries were multiplexed at 8 samples per lane and sequenced in a Hiseq 2,500 Sequencing System (Ilumina) to a depth of 20–30 million reads per sample.

### 2.3. OTX2 knockdown

For CRISPRi experiments, iPSCs bearing a dCas9-KRAB construct under control of the inducible tetO promoter and integrated into the AAVS1 locus (Mandegar et al., 2016) were passaged onto Geltrex (Life Tech) coated plates at 10% confluency. iPSCs were then transduced with lentiviral particles carrying OTX2 gRNAs in the MRP253 backbone. A gRNA sequence targeting the dominant OTX2 transcription start site (GGAAAGTCGGCCCAAATCGG) was sourced from Gilbert et al. (2014). Lentiviral particles bearing the gRNAs were used at a ratio of 1:8 for 24 h in the presence of 10 μM ROCk inhibitor (Y27632, Stratech). Lentiviral media was removed and exchanged for standard Essential 8™ medium (ThermoFisher) for 24 h before being cultured with 1.5 μg/ml puromycin for 24 h. Cells were then cultured in standard Essential 8™. RNA was isolated using standard protocols (Gilbert et al., 2014). CRISPRi iPSCs were plated out, and 2 μg/mL doxycycline (Sigma) added 48 h before iPSCs reached confluence (excluding controls). Once confluent, iPSCs were switched to neural induction medium with or without doxycycline. Neural induction medium comprised 50% Neurobasal medium including B27 and 50% DMEM/F12 supplemented with N2 and Glutamax. The dual SMAD inhibitors 1 μM dorsomorphin and 10 μM SB431542 were also added. Cell media was changed daily and cells in neural induction medium were collected at time points of 0, 1, 2 and 7 days following initiation of neural induction. OTX2 expression was monitored using RT-PCR and RNAseq was carried on an Illumina NovaSeq6000 using 150 bp, PE flow cells. We thank the Oxford Genomics Centre at the Wellcome Centre for Human Genetics (funded by Wellcome Trust grant reference 203,141/Z/16/Z) for the generation and initial processing of sequencing data.’

### 2.4. Real-time polymerase chain reaction (Q-PCR)

The primers were designed with Primer3Plus (v. 0.4.0) software (Supplementary Table S1). Gene sequences were extracted from the NCBI GenBank and the University of California and Santa Cruz (UCSC) genome and bioinformatics browser (Supplementary Table S1). Complementary DNA (cDNA) was synthetized from 1 μg of RNA using M-MLV Reverse Transcriptase enzyme (Promega, M1701) following manufacturer’s instructions. Real-time polymerase chain reaction (Q-PCR) assays were conducted by using iQ Sybr Green supermix (Bio-rad, 178,880) according to the provider specifications. Amplifications were performed in a Bio-Rad PTC-200 Peltier thermal cycler detection system. The housekeeping gene GAPDH was used to normalize the genes expression levels between technical replicates. The Pfaffl comparative method of relative quantification was used to quantify gene relative expression of each sample at different time points. The reference sample used to compare the gene expression of all cell lines was randomly designated as the time point 0 of the 3 technical replicates (Supplementary Figure S2). The means between samples were compared by a one-way ANOVA test with Bonferroni correction with 95% confidence interval with Prism package of GraphPad software.

### 2.5. Immunocytochemistry

Approximately 100,000 cells were plated on each well of Nunclon delta surface 96 well plates (Thermo Fisher148761) and fixed at different time points with 4% paraformaldehyde (PFA) (Thermo Fisher, 28,906) in PBS 1X for 15 min at room temperature. The wells were washed 3 times with 1X PBS and permeabilized and blocked by incubation with 4% normal donkey serum (Sigma Aldrich, D9663) in 1X PBST. The nuclei were stained by incubation with Hoechst 33342 (Thermo Fisher, H3570). Cells were incubated with specific dilutions of mouse monoclonal and rabbit polyclonal primary antibodies together according to the provider specifications (Supplementary Table S2). Validation of the antibodies binding specificity was assessed by replacing primary antibodies with the same dilution of purified mouse IgG (Merck Millipore, CS200621) or rabbit IgG as controls (Thermo Fisher, 02–6,102). Immunoreactivity was analyzed by using Alexa Fluor 594 conjugated donkey anti-mouse IgG (Invitrogen, A-21203) and Alexa Fluor 488 conjugated donkey anti-rabbit IgG (Invitrogen, R37118) both diluted to 1:250 ratio in blocking buffer.

Images were acquired with a 20X objective with the Cell insight CX5 High Content Screen Platform (Thermo Fisher, CX51110). Expression was quantified using the bioapplication Cell Health Profiling from the iDev software package (Thermo Fisher). Hoechst staining was used to assess cell viability. Specific staining intensity, shape and size parameters were established to identify positive and negative labeled cells. A total of 3 wells were analyzed per primary antibody pair with 61 acquired fields per well. The means of the percentages of positive cells at each time point were statistically compared by a two-way ANOVA test with 95% confidence interval with Bonferroni correction with Prism package of GraphPad software.

### 2.6. RNA-seq data analysis

RNA-seq fastq files were aligned to the Human Reference Genome (version hg38) and FPKM counts (Refgene) were generated using the Omicsoft Aligner (OSA; Hu et al., 2012). Only genes with FPKM counts greater than 1 in any sample were included in further analysis. For the time-series data, a gene was called differentially expressed (DE) based on a fold change (FC) cutoff of 2 (up or down) detected at a minimum of 2 time points with respect to time 0. However, because of the availability of replicates in OTX2 knockdown experiment, DESEQ2 with value of p cutoff of 0.05 and FC cutoff of 1.3 was used for DE gene identification. Enrichment analysis was performed using Gene set Enrichment Analysis (GSEA) to identify the biological significance (Subramanian et al., 2005). GSEA was used to avoid the limitation due to 1 replicate and high cut off used for DE analysis in time-series data as it uses the information of all the genes in the enrichment process. Gene Ontology (GO) and Reactome, Kyoto Encyclopedia of Genes and Genomes (KEGG) pathways were used to identify overrepresented annotation terms.

### 2.7. Protein–protein interaction networks

Protein–protein interaction (PPI) analysis was performed using STRING database in Cytoscape.^1^ For the PPI analysis, union of all DE genes from all time points were used. For example, DE genes from all the time points in 48 dataset were combined to generate the 48 h PPI network. PPI edge confidence score of 0.9 and 0.7 were used for TF networks and OTX2 targets networks, respectively. Network modules were identified using Glay method in Cytoscape and annotated manually.

### 2.8. Transcription factor-target analysis

TRANSFAC® data was used initially as the source for Transcription factor (TF)–target gene information. However, curation of TRANSFAC was over-represented by the large number of immune system and disease studies, thus the analysis using TRANSFAC data produced spurious results indicating a major role of JUN, FOS and STATs in neural induction. To overcome this problem, ChIP-seq data on human embryonic stem cells (ESCs) from Tsankov et al. (2015)) were used. Bed files were downloaded from GEO [GSE61475]. Peaks in the promoter region, −2000 – 500 base pair from transcription start site, were annotated using TxDb in R. To identify OTX2 targets important for the neural induction, the targets of OTX2 at 0 h were subtracted from the targets of OTX2 at 120 h, resulting in 1623 targets.

## 3. Results

### 3.1. Phenotypic changes in early neural induction

**Figure 1:**
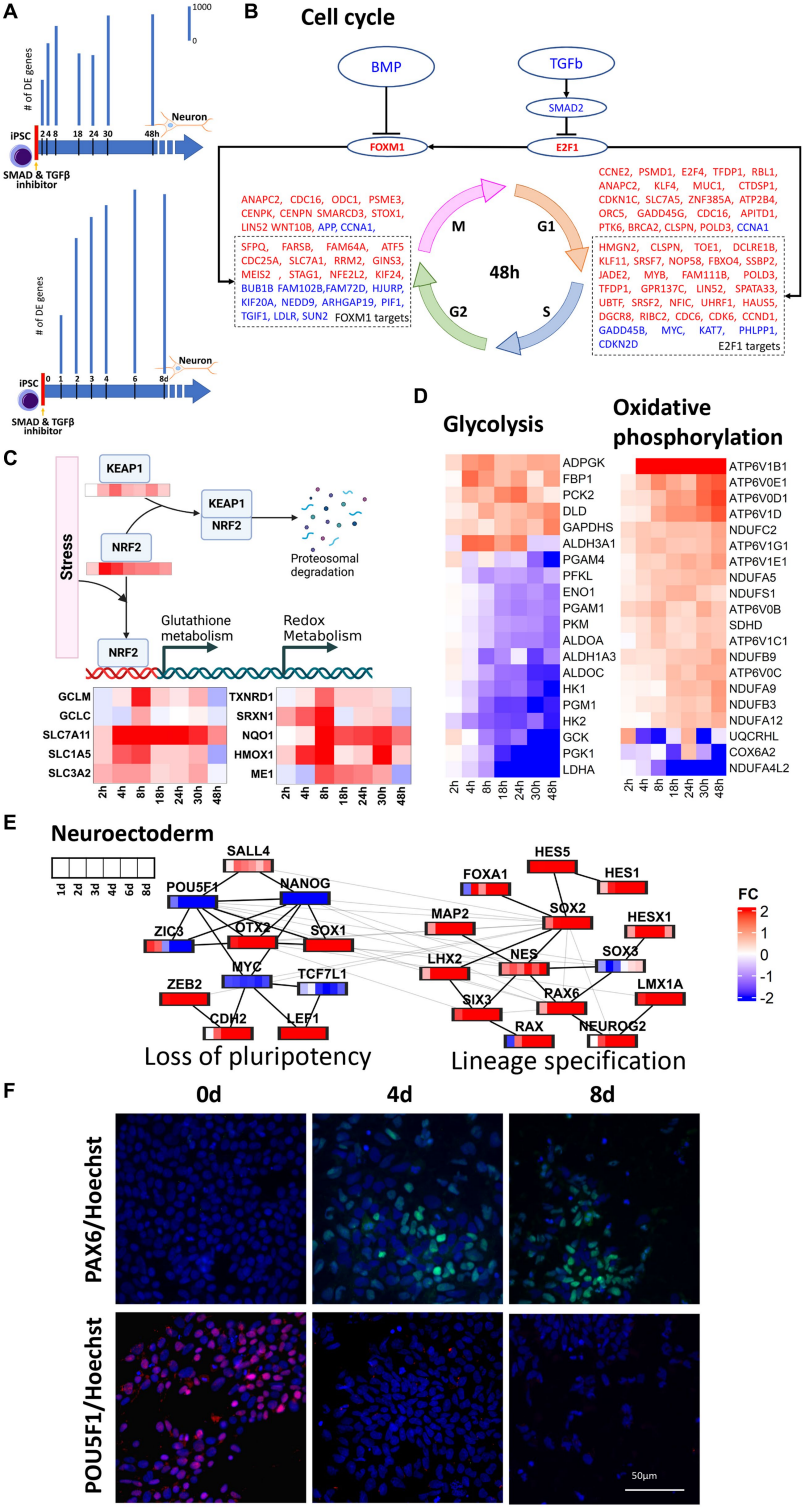
Regulation of key cellular pathways during neural induction. For this analysis we used 1 cell line (CTR_M1_04) and 1 replicate/well. **(A)** represents a schematic of the experimental design and bar plots indicate the number of differentially regulated (DE) genes identified from RNA-seq measurements in two time series, 0–48 h and 0–8 days. Bar plots showing a temporal increase in DE genes. DE genes were computed with respect to their respective expression at d0. **(B)** E2F1 and FOXM1 mediated upregulation of cell cycle in 0–48 h. Upregulation and downregulation of the gene was represented as red and blue font color, respectively. **(C)** NRF2 mediated regulation of oxidative metabolism. **(D)** Heatmaps show the decrease in glycolysis and concurrent increase in oxidative phosphorylation in the 0–48 h dataset. **(E)** protein–protein interaction analysis of pluripotency and neuroectoderm transcription factors using the 0–8d dataset. **(F)** PAX6 and POU5F1 immunocytochemistry analyses of differentiating neural cells. Expression of the neuroectodermal marker PAX6 (top -green) and the pluripotency marker POU5F1 (bottom -red) at time points iPSC, 0d (left), 4d (middle) and 8d (right). The cells nuclei are stained with Hoechst and is shown in blue. The immunocytochemistry statistical analyses shows that the expression of PAX6 significantly increases after 4d of neural induction, whereas POU5F1 is highly expressed in iPSC and is not detected at time points d4 and d8. Three independent replicates/wells for the cell line CTR_M1_04 were used for the immunocytochemical analysis.

In this study, transcriptomic data were harvested throughout 0–48 h and 0–8 days of neural induction of human iPSCs. These datasets were then used to infer the transcriptional regulatory mechanisms operative throughout the early stages of neural induction. Monotonically increasing number of DE genes (Figure 1A) in both time series corroborated the start of the neural induction. In the 0–48 h dataset, upregulation of cell cycle genes was seen (Figure 1B) as evidenced by regulation of cyclins such as CCND1, CCNB1, and CCNE1 and their upstream regulators CDK2, CDK4, and CDK6. The cell cycle master transcriptional regulation factors, E2F1 and FOXM1, genes were also upregulated but did not qualify as DE in our data. E2F1 mainly regulates, G1, S and G1-S phase transition genes such as CDC6, CDK6, RBL1 and CDKN1C. E2F1 but can also directly upregulate FOXM1, which in turn, regulates G2, M, G2-M phase transition genes such as CDC25A, STAG1, MEIS2, and KIF24. Upregulation of E2F1 and FOXM1 can be attributed to the inhibition of TGFB and BMP2 resulting from the use of dual-SMAD inhibition used to initiate neural induction. However, the regulation of cell cycle genes was less prominent in the 0–8 days dataset.

Stem cell differentiation is accompanied by the increase in reactive oxygen species (ROS) and oxidative stress (Hu et al., 2018) and this was supported by the observed increase in NOX4 and NOXA1 in the 0–48 h dataset. Oxidative stress can lead to the stabilization of NRF2 and prevent KEAP1 mediated proteasomal degradation of NRF2 (Lin et al., 2016), which was seen to be upregulated in the 0–48 h dataset. Upregulation of KEAP1 in the 0 -48 h dataset can be attributed to the response to oxidative stress (Baird and Yamamoto, 2020). Upregulation of NRF2 was accompanied by transcriptional regulation of its targets responsible for regulation of distinct aspects of oxidative stress including glutathione metabolism (GCLM, GCLC, SLC7A11, SLC1A5), redox metabolism (TXNRD1, SRXN1), iron metabolism (HMOX1), NADPH production (ME1) and detoxification (NQO1), as seen in 48 h dataset (Figure 1C). Glutathione metabolism also plays a critical role in maintaining genomic integrity, which is essential for neural induction (Chui et al., 2020). Increases in expression of glutamine transporters further lead to increase in mitochondrial oxidative phosphorylation and this increase in oxidative phosphorylation was inferred from the increase in NADH dehydrogenases (NDUFC2, NDUFA5, NDUFA9 and NDUFA12) and ATPases (APT6V1B1, ATP6V1D, ATP6V0D1 and ATP6V0E1). Though glycolysis is a major source of energy required to maintain stem cell functions, early differentiation of human pluripotent stem cells (hiPSC) has been reported to be accompanied by a switch from glycolysis to oxidative phosphorylation (Zhang et al., 2012; Zheng et al., 2016) owing to the increased energy demands. Our data supported the decrease in glycolysis and increase in oxidative phosphorylation within 48 h of initiation of neural induction (Figure 1D). In addition to increases in glutamine uptake and glutathione metabolism, a reciprocal down-regulation of glucose transporter (SLC2A1), glycolysis gate keepers (HK1 and HK2), glycolysis pathway genes (PGK1, PGM1, ENO1 and PKM) and lactic acid utilization genes (LDHA) was also observed. Taken together, these observations showed a dynamic regulation of multiple transcriptional modules during the cell state transition from iPSC through neural induction to neuroectodermal stem cell neural that couple gain of neural identity and loss of pluripotency with changes in key cellular processes including regulation of cell cycle, metabolism and stress response.

To understand the temporal transcriptional regulation of neural induction, knowledge-based lists of pluripotency genes, neural induction transcription factors and neuroectoderm markers were curated (Figure 1E) and analyzed based on protein–protein interactions (PPI, see method section). For PPI analyses, union of DE genes across all time points were taken. 0–48 h and 0–8 days datasets identified 3,893 and 6,071 genes, respectively. PPI analysis of the 0–48 h dataset identified a loss of pluripotency module, as demonstrated by the staged loss of pluripotency markers, ZIC3, NANOG and POU5F1 (Figures 1E,F; Supplementary Figure S4), as early as 8 h after initiation of neural induction, with subsequent loss of LCK and KLF5 from 2d (Figure 1E) accompanied by a reciprocal increase in neural stem cell markers NES and SOX1. In the 0–8 days dataset, in addition to loss of pluripotency, a neural lineage specification module was identified, characterized by up-regulation of several key transcription factors known to regulate both transcription and chromatin dynamics during neural induction, including OTX2, SOX2, RAX, LHX2 and SIX3 (Figure 1E). Another key regulator of both pluripotency and neural induction, SOX2 was also identified as a key regulator based on protein interaction analysis, while up-regulation of WNT regulators, LEF1 and ZEB2 (Supplementary Figures S3, S4), and increasing expression of neuroectoderm markers, PAX6, NEUROG2 and LMX1A (Figures 1E,F), underscored the transition of iPSCs toward the neural lineage. The outcome of these PPI analyses thus underscored the tight coupling of lineage specification and regulation of key cellular processes seen in our analysis of DE genes.

### 3.2. Global interaction network of transcription factors

**Figure 2:**
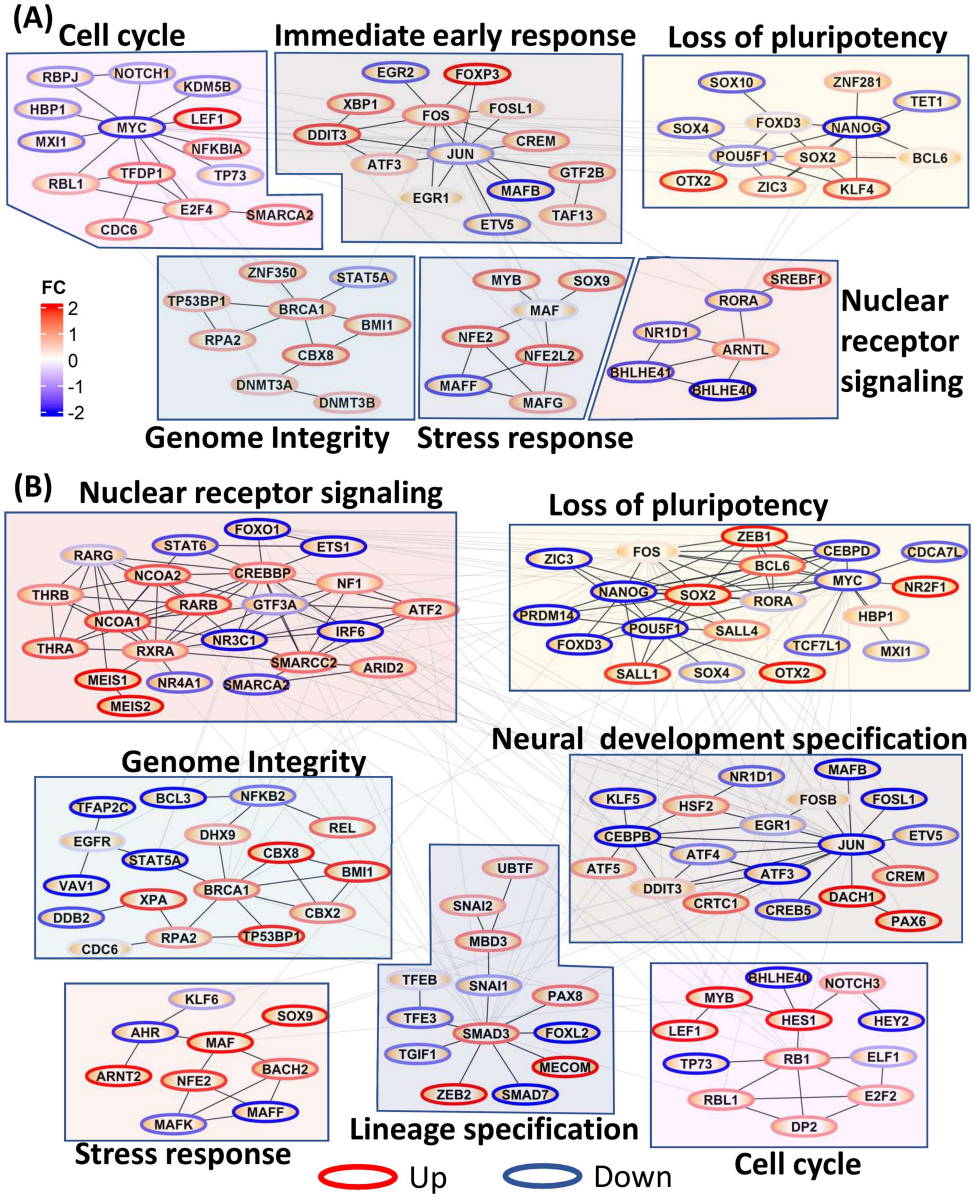
Protein–protein interaction (PPI) network of all transcription factors. Network modules were identified using Glay method in Cytoscape and annotated manually. Node borders were colored red and blue based on the average fold change of the TF across all time points. **(A)** PPI network showing the increase in cell cycle, immediate early gene response, stress response, genome integrity, and loss of pluripotency in 0–48 h dataset. **(B)** PPI network showing increases in nuclear receptor signaling, genome integrity, stress response, lineage specification, loss of pluripotency and neural development specification in the 0–8d dataset.

We further constructed PPI networks using all DE TFs to provide a global view of the dynamic changes in transcriptional regulation throughout neural induction. In 0–48 h dataset (Figure 2A), upregulation of TFs associated with immediate early response and stress response was seen, probably as a consequence of the dual SMADi initiation of neural induction. Moreover, in addition to regulation of stress response, MAF genes also play a role in cortical development (Pai et al., 2020). Further, several nuclear receptor TFs, known to be regulators of stemness were also downregulated during this time period. Cell cycle regulatory TFs (CDC6, LEF1, NKFB1A, RBL1, TFDP1, SMARCA2) were seen to be upregulated, as observed earlier. Though MYC is downregulated, our data show that E2F1 and FOXM1 which drive early phases of cell cycle are upregulated. In addition to these modules, an increase in TFs associated with genome integrity and genome stability (DNMT3A, DNMT3B, BRCA1, BMI1, TP53BP1, RPA2) was observed, congruent with the maintenance of stemness during differentiation (Li et al., 2020) while, a module of pluripotency genes was reciprocally down-regulated.

Many of the functional modules identified in the 0–48 h time series were also evident in the 0–8 days time series, but strikingly the TF membership of the modules was only partially conserved (Figure 2B). Thus, pluripotency maintenance genes, which were downregulated in 0–48 h dataset, were further downregulated in the 0–8 days timepoints. In addition to the decrease in expression of canonical pluripotency factors, POU5F1 and MYC, seen at 0–48 h, downregulation of their target genes CEBPD, MXI1, PRDM14, and CDCA7L was also evident, indicating a consolidated loss of pluripotency. ZIC3, upregulated at 0–48 h and required for maintenance of pluripotency, was downregulated from 3d onwards at the onset of neuroectodermal differentiation (Morrison and Brickman, 2006). In 0–8 days, neural stem cell markers DACH1 and PAX6 were upregulated indicating formation of neuroectodermal stem cells while HSF2, a cortical developmental marker, was also upregulated in 0–8d (Chang et al., 2006) congruent their cortical identity. Concurrent upregulation of TFEB, PAX8, ZEB2, and SNAI2 is consistent with maintenance of a precursor/multipotent state prior further differentiation of these cells toward neuronal and glial lineages (Yuizumi et al., 2021).

### 3.3. Characterization and validation of OTX2 role in early neural induction

Our studies identified OTX2 as one of the most precociously expressed transcription factors during neural induction (Supplementary Figures S5A,B). OTX2 is known to play a pivotal role during three distinct stages of neurodevelopment, including (i) maintenance of the naiive pluripotent ground state and for transition to the primed pluripotent state (Yang et al., 2014) (ii) coordinate repression of pluripotency and activation of neural induction (Greber et al., 2011; Malchenko et al., 2014) (iii) control of rostral fate specification of telencephalon and mesencephalon (Simeone, 1998; Simeone et al., 2011). Furthermore, OTX2 has been suggested to act as a pioneer factor (Boulay et al., 2017) indicating its potential as a driver of state transition. This convergence of precocious temporal expression and known functionality suggested that manipulation of OTX2 expression would be an appropriate test to validate our model of dynamic transcriptional regulation during neural induction. TRANSFAC® data was initially used as the source for TF-target gene information. However, curation of TRANSFAC was over-represented by the large number of immune system and disease studies such as cancer. Accordingly, we were concerned that analysis using TRANSFAC data could produce spurious results such as indicating a major role of JUN, FOS and STATs in neural induction. To overcome this problem, ChIP-seq data on POU5F1, NANOG and OTX2 derived from human embryonic stem cells (ESCs) from Tsankov et al. (2015) were used to more faithfully recapitulate TF-target gene interactions in human iPSCs. OTX2 chip-seq produced 1,623 targets genes. The intersect of OTX2 targets genes and the combined gene lists of 0–48 h and 0–8d dataset, used for PPI analysis, resulted in 310 and 507 genes. PPI analysis of these 310 and 507 genes emphasized the role of OTX2 in cell cycle specially speckle formation and spliceosome function as evidenced by upregulation of SRSF1, SRSF2, SRSF7, FUS and SF1 in the 48 h dataset and SRSF1, SRSF2, FUS and DHX9 in the 0–8d dataset, indicating a direct role of OTX2 in regulation of the spliceosome machinery. In addition to upregulation of spliceosome, ribosome genes (RRS1, RPF2, and METTL1) were also upregulated in 0–48 h dataset and notch signaling (NOTCH2, SEL1L, and DLL1) was upregulated at 0–8d dataset.

**Figure 3:**
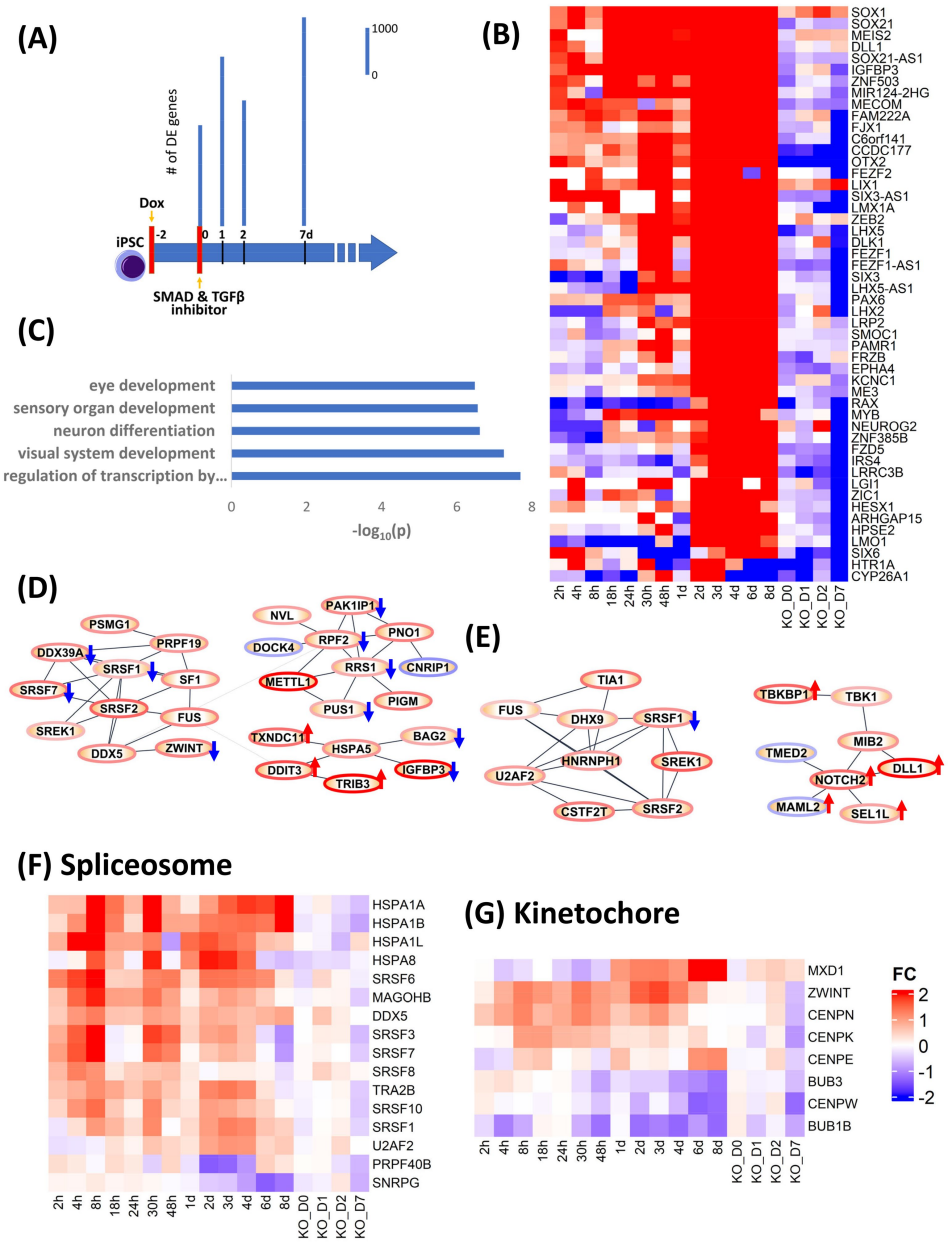
Validation of role of OTX2 in neural induction using CRISPRi KD of OTX2. **(A)** schematic of experimental design. Doxycycline (Dox) was used to initiate the KD of OTX2 for 48 h prior to neural induction. Bar plots show differentially regulated (DE) genes from RNA-seq measurements in the time points, 0, 1, 2, and 7d. DE genes were computed with respect to same time Dox^−^ control point. **(B)** Heatmap of the top 50 downregulated genes to show the effect of OTX2 KD. **(C)** Barplot of GO biological processes enrichment of top 50 downregulated genes in OTX2 KD. **(D,E)** PPI network of OTX2 selected targets based on Tsankov et al. (2015) in the 0–48 h and 0–8d datasets, respectively. Node border colors, red and blue, show average up- and down- regulation, respectively, of the genes. The effect of OTX2 KD is shown as red (up) and blue (down) arrow on the nodes. **(F,G)** Heatmaps show the downregulation of spliceosome and kinetochore pathway genes.

In order to validate the causality implied by our analysis of the dynamic transcriptional landscape during neural induction, we used CRISPRi to knock down (KD) expression of OTX2 (Supplementary Figure S5C), indicated by our analysis above to be involved in regulation of several key cellular processes during neural induction and one of the most precociously expressed transcription factors during neural induction (Acampora et al., 2013). Doxycycline (Dox) was used to induce expression of a dCas9-KRAB construct to inhibit expression of OTX2 for 48 h prior to neural induction. Samples were collected for RNAseq at 0, 1, 2 and 7 days. Control samples (DOX-) were also collected at the same time (Figure 3A). Data were analyzed by calculating fold change in gene expression for OTX KD versus WT at each time point. To analyze the effect of OTX2 KD, the top 50 downregulated genes (Figure 3B) were selected based on ranking of mean of FC of 0–8d dataset minus FC of KD at D7. Enrichment of these 50 genes (Figure 3C) indicated a disruption of neural induction. Known targets of OTX2 such as PAX2 and LHX2 were downregulated in top 50 genes while other neural differentiation genes including LHX5, LMX1A and NEUROG2, involved in neuronal patterning, were also down-regulated. Forty two cell cycle genes were downregulated in OTX2 KD, while cell cycle inhibitors (RB1, SMAD3 and CDKN1A) were upregulated thus indicating overall suppression of cell cycle. Downregulation of spliceosome genes, SRSF3, SRSF6, SRSF8, and SRSF10 was also observed (Figures 3D–F), thus conforming our hypothesis on the role of OTX2 in regulation of splicing. Furthermore, kinetochore genes, CENPN, CENPE, CENPK, and BUB3, were also downregulated (Figure 3G). This downregulation of spliceosome and kinetochore genes further supports the downregulation of cell cycle. Interestingly, the regulation of notch and cAMP signaling pathway was further enhanced compared to WT (Figure 3E; Supplementary Figure S6). Importantly, the effects of CRISPRi KD of OTX2 corroborate the regulatory modules inferred from our DE gene and PPI analyses.

## 4. Discussion

During the course of cortical neural induction, iPSCs go through a series of cell states, starting from the pluripotent stem cells and culminating in multipotent neuroectodermal stem cells, which then undergo further maturation before terminal differentiation into cortical neurons. Our results show the transcriptional and regulatory mechanisms that are associated with this transition across time. BMP signaling is required for the maintenance of pluripotency in human stem cells (Osnato et al., 2021) and blockade of BMP signaling using dual SMAD inhibiton has become the standard protocol for inducing neural induction toward early neuroectoderm (Tomishima, 2012). Our study shows precise transcriptional regulatory mechanisms that orchestrate this induction process in a temporally relevant manner. In parallel we differentiated iPSC using WNT inhibition for 7 days to determine if the molecular events underlaying neuronal differentiation are significantly altered by using an alternative neural induction protocol. The results indicate that WNT inhibition does not significantly alter the gene expression patterns during the initial 8 days of neural induction (Supplementary Figure S1).

We show, as expected, the suppression of canonical stemness TFs, POU5F1, and NANOG beginning as early as day 1, continuing into day 8, while lineage-altering factors ZIC2, SOX2, and KLF4 increased in expression over this time period. Interestingly, during the 48 h time frame, in addition to loss of pluripotency, we saw regulation of immediately early response genes jun-fos signaling, genes that are responsible for maintaining genome integrity during the rapid alterations in chromatin topology and expression of key stress response genes. Loss of pluripotency is strongly coupled to stress response, as evidenced by the induction of KEAP and NRF2 genes, precursors to the pathway that is a principal protective response to oxidative and other stresses (Baird and Yamamoto, 2020). We also found that NRF2 regulates transcription of several stress response genes as seen in our analysis of the 0 – 48 h dataset, with KEAP serving as the sensor. Further KEAP1-NRF2 pathways promote metabolic reprogramming *via* control of central carbon metabolism through the pentose phosphate pathway and glutamine metabolism (Lin et al., 2016; Sayin et al., 2017).

Further, repression of the stemness genes concomitantly led to suppression of pluripotency maintenance genes while activating expression of several zinc finger genes responsible for inducing changes in chromatin topology with concomitant changes in the cell state. Our results also show that in the initial 48 h time frame, several cell cycle processes are robustly activated at each phase of the cell cycle implying the progression from pluripotency. Moreover, we observed repression of several nuclear receptor superfamily genes, establishing the temporal links between pluripotency and the NR superfamily TFs, which are known to be required for both maintenance and suppression of pluripotency (Jeong and Mangelsdorf, 2009; Wagner and Cooney, 2013). While loss of pluripotency is initiated in within 48 h of the induction process, it persists for 8 days while robustly activating the expression of SOX2 and SALL4 genes, which crosstalk with the stemness TFs to coordinate activation of the transcriptional framework that concurrently represses stemness genes while activating early neural induction genes. This tightly coupled repression of pluripotency and activation of neural induction was reflected in the transcription factor complex comprising POU5F1, SOX2, NANOG, MYC, SALL2, SALL4, CTNNB1, OTX2, and LKF4, all of which were regulated during the 8 day neural induction period. This tight transcription factor complex functions to orchestrate the Janus face of the cell fate, namely concurrent loss of pluripotency and the activation of neural induction. Our perturbation using CRISPRi to knock-down OTX2 expression sharply illuminated the functional and temporal complexity of this complex. While broadly the OTX2 knock-down did not alter the cell fate, it did change the kinetics of the induction process, manifested as a delay in the repression of pluripotency and a disruption of neural induction, accompanied by downregulation of several components of spliceosome machinery, and several kinetochore genes, the latter resulting in disruption of the normal kinetics of the cell cycle.

**Figure 4:**
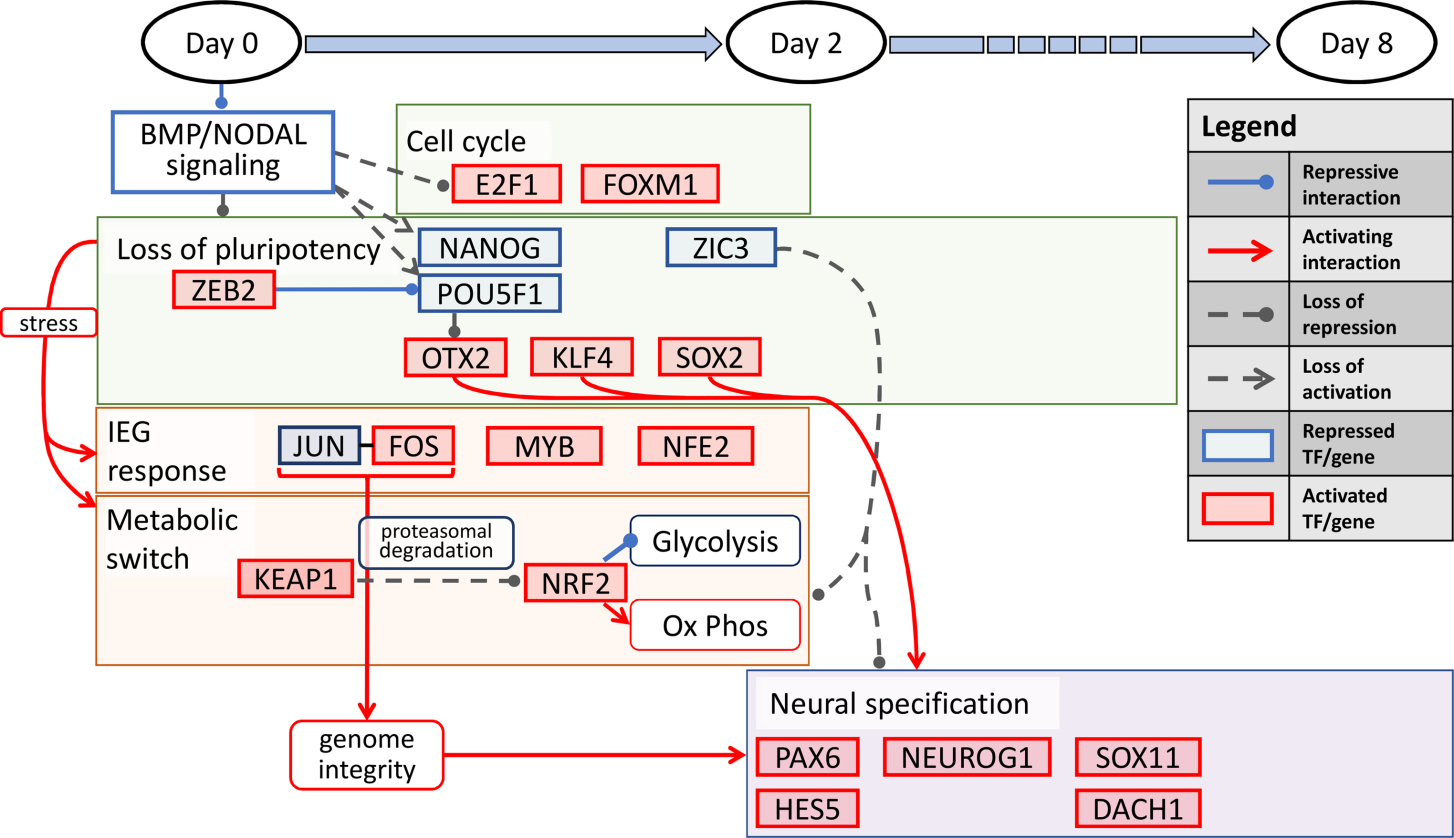
A temporally-associated regulatory network for neural induction. Dual SMAD inhibition causes an increase in cell cycle *via* upregulation of E2F1 and FOXM1 accompanied by the loss of pluripotency due to repression of NANOG and POU5F1. The loss of pluripotency in turn leads to neuronal induction *via* OTX2, KLF4 and SOX2. In addition, stress induced IEG response and metabolic switch from glycolysis to OXPHOS further allude to the increase in genome integrity and neuronal specification throughout neural induction.

Our results show an interesting interplay among several processes precisely orchestrated in a temporally defined manner during neural induction. The overall transition can be summarized into three distinct stages. First, during the temporal dynamics spanning the entire 8-day period, the cell state changes dynamically manifest as loss of pluripotency and a gain of neural identity. This process is defined by repression of the pluripotency transcription factors, POU5F1 and NANOG, and activation of KLF4, along with regulation of other pluripotency and chromatin state modifiers, OTX2, HDAC1, ZIC3, and ZEB2. The latter two are zinc finger factors known to alter the chromatin topology in the pluripotent state to a lineage state (Kwak et al., 2018). Second, In the first 2 days of neural induction, the key processes that accompany the loss of pluripotency include immediate early response, processes for maintenance of genome integrity, and induction of a metabolic switch from a predominantly glycolytic state to a more oxidative state. The key transcription factors that account for these processes include, JUN, FOS, CREM, NFE2, MYB, NRF2, and KEAP1, the latter of which serves to induce the metabolic switch. Third, in the transition from the early lineage state in the 2 day post neural induction period to d8, the key process is progression to a neuroectodermal state, orchestrated by the canonical neural factors, PAX6, NEUROG2, SOX11, DACH1, ASCL1, and HES5 (Ozair et al., 2013). We illustrate our overall schematic of temporally defined neural induction processes in Figure 4.

The above mechanisms pose interesting questions on the lineage specification in neurological disorders of genetic origin such as familial Alzheimer’s disease (FAD) induced by specific mutations in PSEN1, PSEN2 or APP proteins. It has been established that transcription factors NRF2 and REST play a crucial role in defining cell fate outcomes in FAD (Caldwell et al., 2020, 2022). Not surprisingly, the endpoint lineage in these iPSC-derived diseased neurons is dedifferentiation to neuronal precursor states and reduced expression of neuron lineage genes. Our study has implications for understanding neurodevelopmental disorders as well as neurodegeneration. Toward addressing the former, our study recommends targeted perturbations to enhance specific neural induction stages or repress progression to other lineages. In our study on neurodegenerative disorders, we demonstrated that neurons dedifferentiate into distinct precursor-like stages offering potential interventions to reinitiate progression into the neuronal stages (Caldwell et al., 2020, 2022). A longer study exploring this lineage specification to the 50 day point when the neuronal precursors become mature is in progress.

Much of our understanding of the gene regulatory pathways underlying the initial steps of neuronal development is largely based on animal models, particularly in Xenopus and mouse. We summarize here, some of the most salient observations. In Xenopus, Foxd4 is expressed in the neuroectodermal precursors and promotes the expression of Gmnn, Sox11 and Zic2. Foxd4, Gmnn, Zic2 and Sox11 block non-neural induction by inhibiting BMP/WNT pathways and their targets (Klein and Moody, 2015). The default neural induction model suggests that inhibition BMP and TGFβ is sufficient to induce neural differentiation in the competent ectoderm by preventing expression of the epidermal and mesodermal transcription network promoted by BMP and TGFß, respectively (Marchal et al., 2009; Andoniadou and Martinez-Barbera, 2013; Klein and Moody, 2015) SoxB1 family members Sox1, Sox2 and Sox3 are up-regulated downstream of Foxd4, Gmnn and Zic2 and are necessary to maintain self-renewing progenitors and stablish spatial identity of neural cells. Additionally, SoxB1 factors maintain neuroectodermal fate by inhibiting expression of Vent2, a BMP target necessary for epidermis differentiation (Rogers et al., 2009; Lee et al., 2014). Zic1, Zic3, Irx1-3 are up-regulated upstream of early bHLH pro-neural factors and are required to maintain neural progenitors in a proliferative state and for temporarily inhibit neural differentiation genes (Aruga and Mikoshiba, 2011; Moody et al., 2013; Sankar et al., 2016). Subsequently, activation of bHLH family factors is required for transition of neuroectodermal cells to neural progenitors (Kageyama et al., 2005; Dhanesh et al., 2016).

Studies in human iPSCs have enabled identification of important markers and drivers of neural differentiation including PAX6, ZEB2, ZNF521, NESTIN and NR2F2 (Ardhanareeswaran et al., 2017). In humans, PAX6 is sufficient and necessary for neural induction possibly by repressing pluripotency genes such as OCT4, NANOG, and MYC. However, OCT4, NANOG and SOX2 knockdown in ESCs induces trophoblastic fate suggesting that inhibition of pluripotency genes is a pre-requisite but not sufficient for neuroectodermal differentiation. It has been proposed that neuroectodermal differentiation is potentiated by PAX6 activation of neuronal progenitor genes including SIX3, LHX2, FGF8, NR2F2, TBR2, and WNT5b (Zhang et al., 2010; Blake and Ziman, 2014). Similarly, it has been suggested that ZEB2 is necessary for neuroectodermal progression and maintenance by antagonizing Activin/Nodal and BMP signaling by direct interaction with SMAD proteins (Conidi et al., 2011; Tang et al., 2015). ESC/iPSC studies have demonstrated that up-regulation of neuroectodermal markers PAX6, SOX1, NCAM, ZIC1, and ZEB2, and loss of pluripotency/self-renewal markers OCT4, NANOG, and KLF4 occurs at day 5–7 after hPSCs induction (Kamiya et al., 2011; van de Leemput et al., 2014; Huang et al., 2016). Subsequently, chromatin modification genes such us TET2, KATB2, and SIRT1 were differentially expressed after 6 and 10 days of neural induction (Huang et al., 2016). These studies, and others, have provided an overview of the molecular factors required for the specific spatiotemporal regulation of neural differentiation. However, the dynamic genetic and regulatory programs underlying the progression from pluripotency state to neural competence remain to be investigated. Here, we provide a unique insight into the initial molecular events that govern the transition of iPSCs to neuroectodermal fate and subsequently contribute to the identification of relevant disease processes. Overall, our study of the dynamics of transcriptional changes during neural induction points to the tight coupling that occurs between transcriptional modules that determine changes in cell state during the transition from pluripotent stem cell to multipotent neural stem cell. This coupling occurs not only at the level of lineage specification but also in parallel regulation of modules that regulate key cellular processes including cell cycle, cellular stress and metabolism. This insight clearly illustrates the importance of taking a dynamic and holistic perspective of cell state transitions to account for the wholesale changes in transcriptional dynamics and cellular rewiring that represents changes in cell state. We anticipate that further studies will dissect further the contribution of other key TFs toward governing the cell state transitions during neural induction.

## Data availability statement

The original contributions presented in the study are publicly available. Data is submitted in GEO repository. This data can be found here: https://www.ncbi.nlm.nih.gov/geo/query/acc.cgi?acc=GSE223624 accession ID: GSE223624.

## Author contributions

SG: data analysis and drafting of manuscript. LP: data acquisition. MF: data acquisition and analysis. HR: data acquisition and critiquing of manuscript. DM: data analysis. NB: experimental design and interpretation, drafting, and critiquing of manuscript. SS: data analysis and interpretation, drafting, and critiquing of manuscript. All authors contributed to the article and approved the submitted version.

## Funding

The authors wish to acknowledge funding support from NIH grants, R01 LM012595 (SS), OT2 OD030544 (SS), R01 HL106579-07 (SS) and the Joan and Irwin Jacobs endowed professorship (SS), SENESCYT PhD scholarship (LP), and Mano Maurya in the Subramaniam laboratory for discussions on analytical methods used.

## Conflict of interest

The authors declare that the research was conducted in the absence of any commercial or financial relationships that could be construed as a potential conflict of interest.

## Publisher’s note

All claims expressed in this article are solely those of the authors and do not necessarily represent those of their affiliated organizations, or those of the publisher, the editors and the reviewers. Any product that may be evaluated in this article, or claim that may be made by its manufacturer, is not guaranteed or endorsed by the publisher.

## Footnotes

^1^https://cytoscape.org
