# Supplementary material for: Temporal transcriptional control of neural induction in human induced pluripotent stem cells
Source: Front Mol Neurosci. 2023 May 5;16:1139287. doi: 10.3389/fnmol.2023.1139287 (PMC10195998; doi:10.3389/fnmol.2023.1139287)
Supplement: Supplementary file 1 [file Data_Sheet_1.docx]

**Supplementary Material**

**Temporal transcriptional control of neural induction in human induced pluripotent stem cells.**

Shakti Gupta^1†^, Lucia Dutan Polit^2†^, Michael Fitzgerald^1^, Helen Rowland^3^, Divya Murali^1^, Noel J Buckley^3*^, Shankar Subramaniam^1,4*^

^1^ Department of Bioengineering, University of California San Diego, San Diego, CA, USA

^2^ Maurice Wohl Clinical Neuroscience Institute, King’s College London, UK

^3^Department of Psychiatry, University of Oxford, UK

^4^Departments of Computer Science & Engineering, and Cellular & Molecular Medicine, University of California San Diego, San Diego, CA, USA

^†^equal contributing and first authorship

^*^equal contribution and senior authorship and corresponding authors


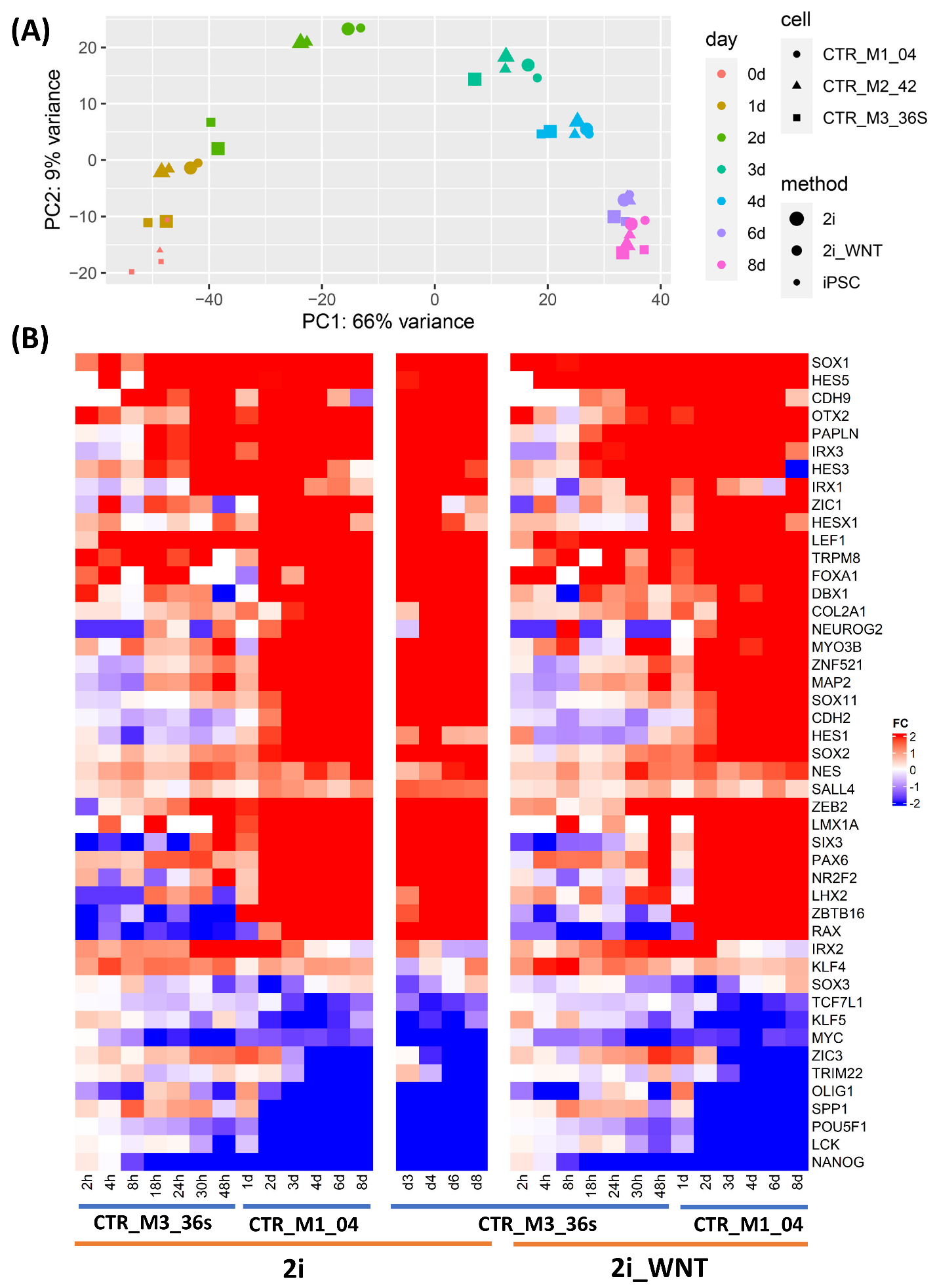


**Fig S1.** Comparison of different neural induction protocols in different cell lines using RNA-Seq data. A) Principal component analyses of 3 iPSC lines induced with 2i or 2i-WNT at all time points. For the PCA we used the iPSC cell lines CTR_M1_04, CTR_M2_42 and CTR_M3_36S with and without the inclusion of the WNT inhibitor XAV939 we used one replicate/well per cell line per time point. PC1 represents 69% of the variance across all samples and is plotted in the axis-x while PC2 represents 9% of the variance and is plotted in the axis-y. Samples from individual iPSCs are show with different symbols. The size of the symbol indicates the method used for differentiation while the time points used for RNA extraction during NESC differentiation are shown in different colours. B) Heatmaps of pluripotency and neuroectoderm transcription factors shows the similarity different cell lines and different neural induction protocols at the gene levels. Upregulation and downregulation of the gene was represented as red and blue font color, respectively.


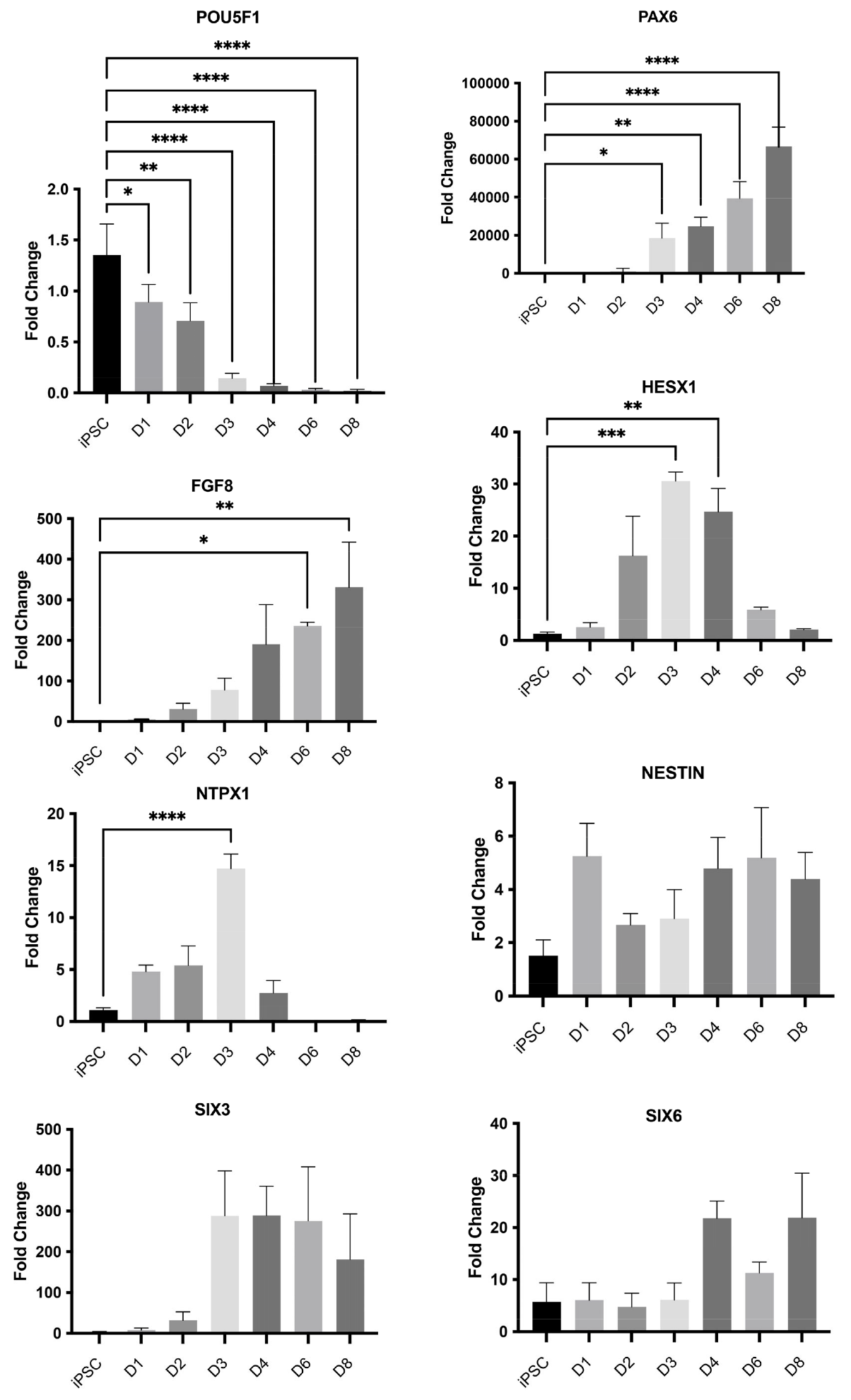


Fig S2. Real time PCR analysis during neuroectoderm differentiation. The Q-PCR data shows the temporal expression patterns of eight genes at time points iPSC, d1, d2, d3, d4, d6 and d8. X-axis indicates the time point of RNA extraction and the Y-axis the fold changes in expression. The pluripotency gene POU5F1 (top) is significantly downregulated after one day of 2i induction and continues to decrease until day 8. The expression of the neuroectodermal makers PAX6 (second) and FGF8 (third) are significantly upregulated after 2 and 6 of 2i induction respectively. The expression of HESX1 (fourth) and NPTX1 (fifth) peaks at day 3 after induction, whereas NESTIN (sixth), SIX3 (seventh) and SIX6 (eight) do not show significant changes in their expression. 3 independent replicates/wells for the cell line CTR_M1_04 were used for the PCR a analysis.


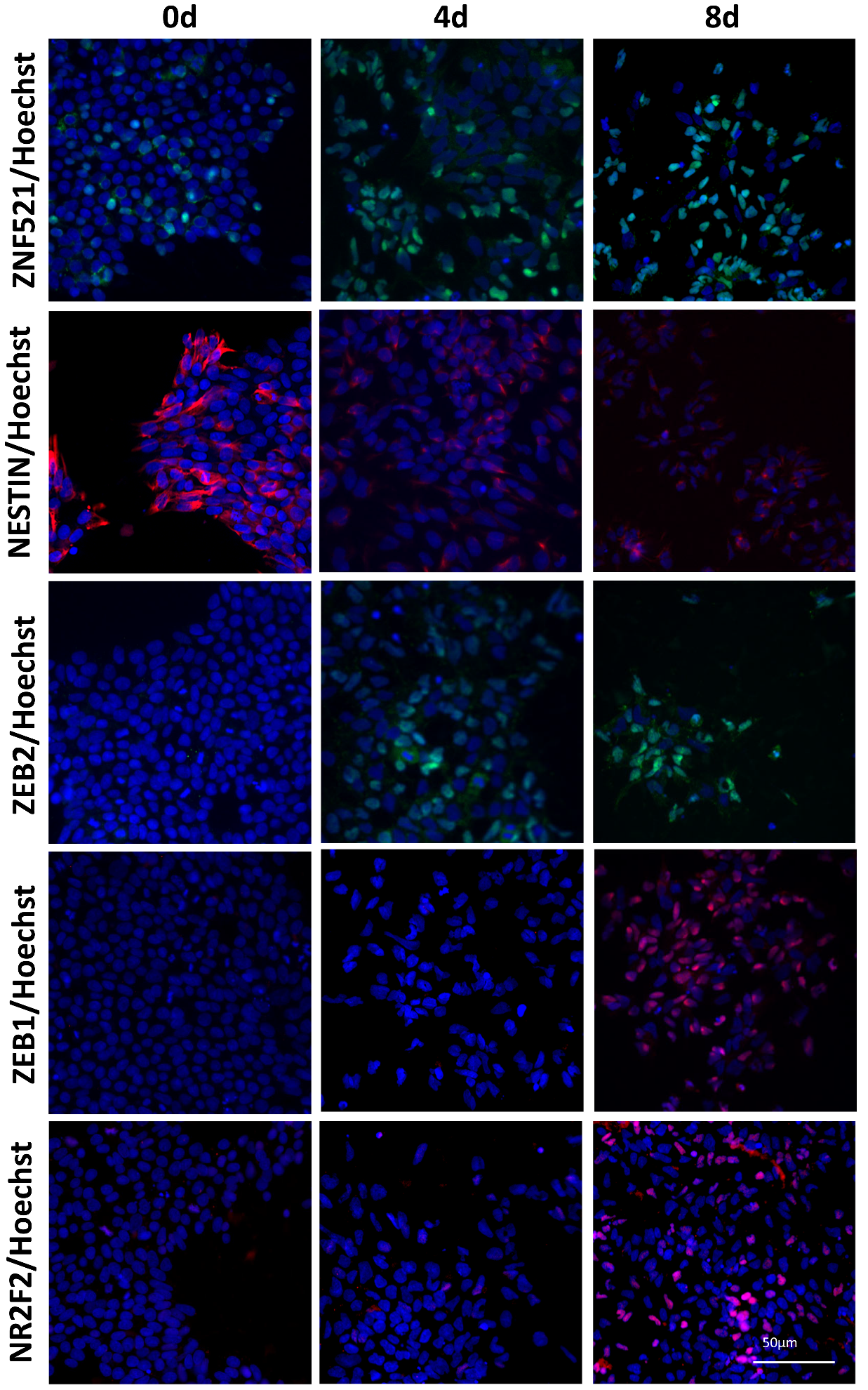


**Fig S3**. **ZNF521, NESTIN, ZEB2, ZEB1, NR2F2** **immunocytochemistry analyses of differentiating neural cells**. The analysis was performed at time points iPSC (left), d4 (middle) and d8 (right). The differentiating cell nuclei is shown in blue. The expression of the neuroectodermal markers ZNF521(top -green), ZEB2 (third -green), ZEB1 (fourth from top -red) and NR2F2 (bottom -red) is low or negative in iPSC and significantly increases after 4 and 8 days of neural induction, whereas NESTIN (second -red) is highly expressed at all time points.


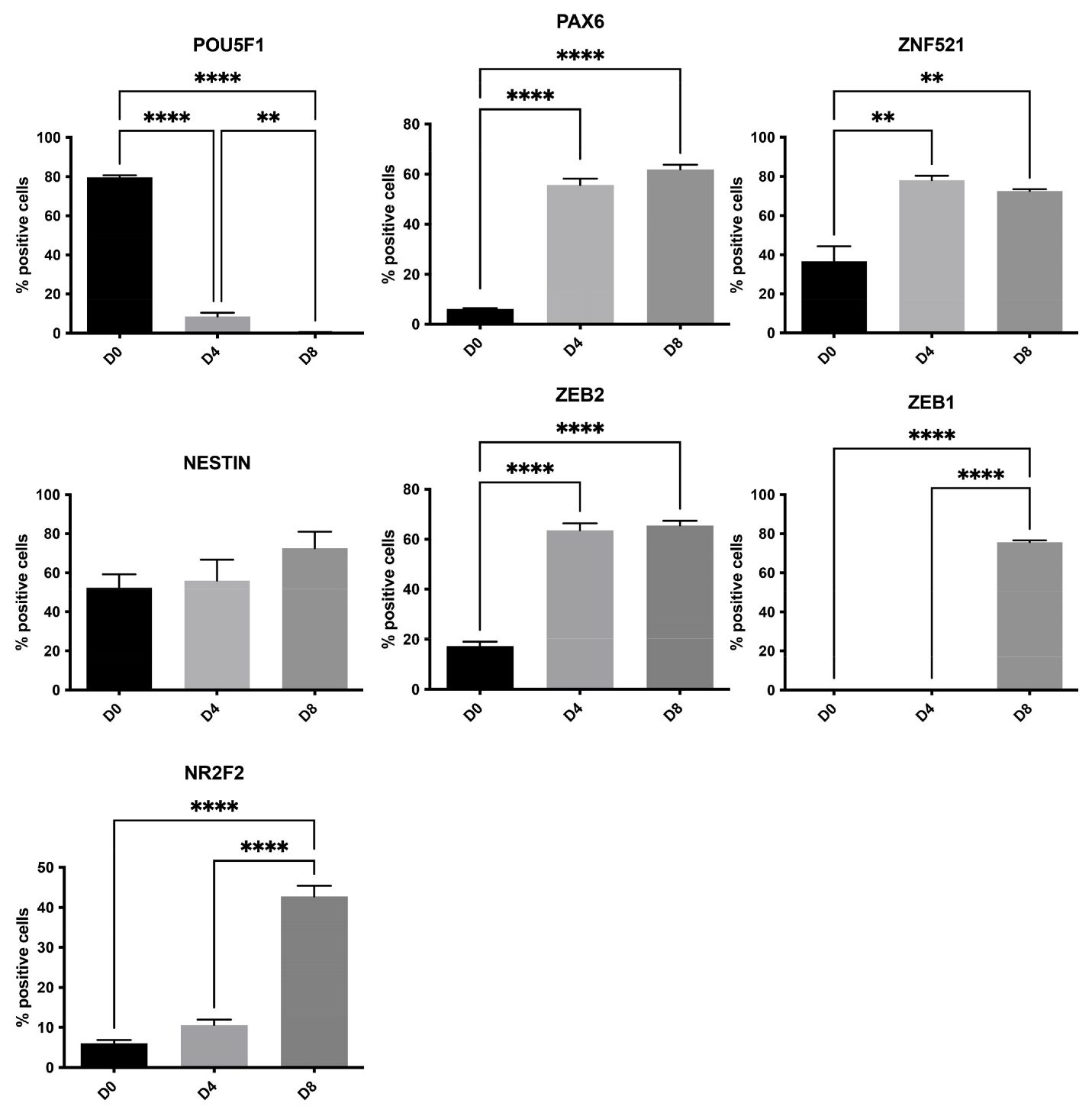


**Fig S4**. **Quantification of POU5F1, PAX6, ZNF521, NESTIN, ZEB2, ZEB1, NR2F2** **immunocytochemistry staining of differentiating neural cells**. The analysis was performed at time points d0 (iPSC), d4 and d8. The percentage of cells expressing pluripotency marker, POU5F1, is high in iPSC and significantly decreases at time points d4 and d8. The percentage of cells expressing positive neuroectodermal markers, PAX6, ZNF521, ZEB2, ZEB1 and NR2F2, is low in iPSC and significantly increase after 4 and 8 days of neural induction, whereas NESTIN is highly expressed at all time points. Error bars indicate standard deviation.


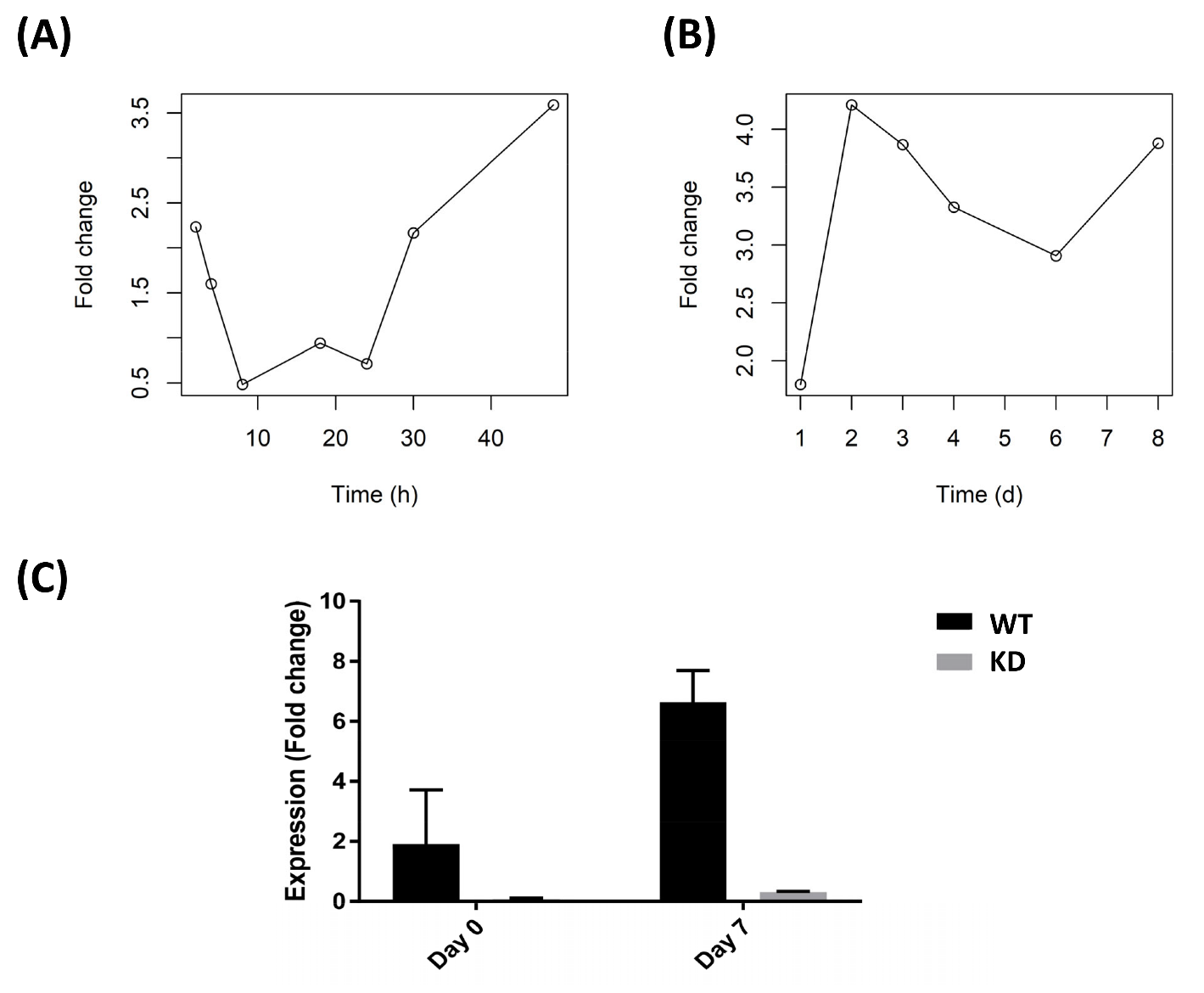


**Fig S5. Characterization and validation of OTX2 role in early neural induction.** A) and B) show the kinetic changes in OTX2 profile in 48h and 8d RNAseq datasets. C) shows the qPCR validation of OTX2 expression in wild type (WT) and CRISPRi knockdown (KD) condition at day 0 and 7. Doxycycline (Dox) was used to initiate the KD of OTX2 for 48 hours prior to neural induction at day 0.


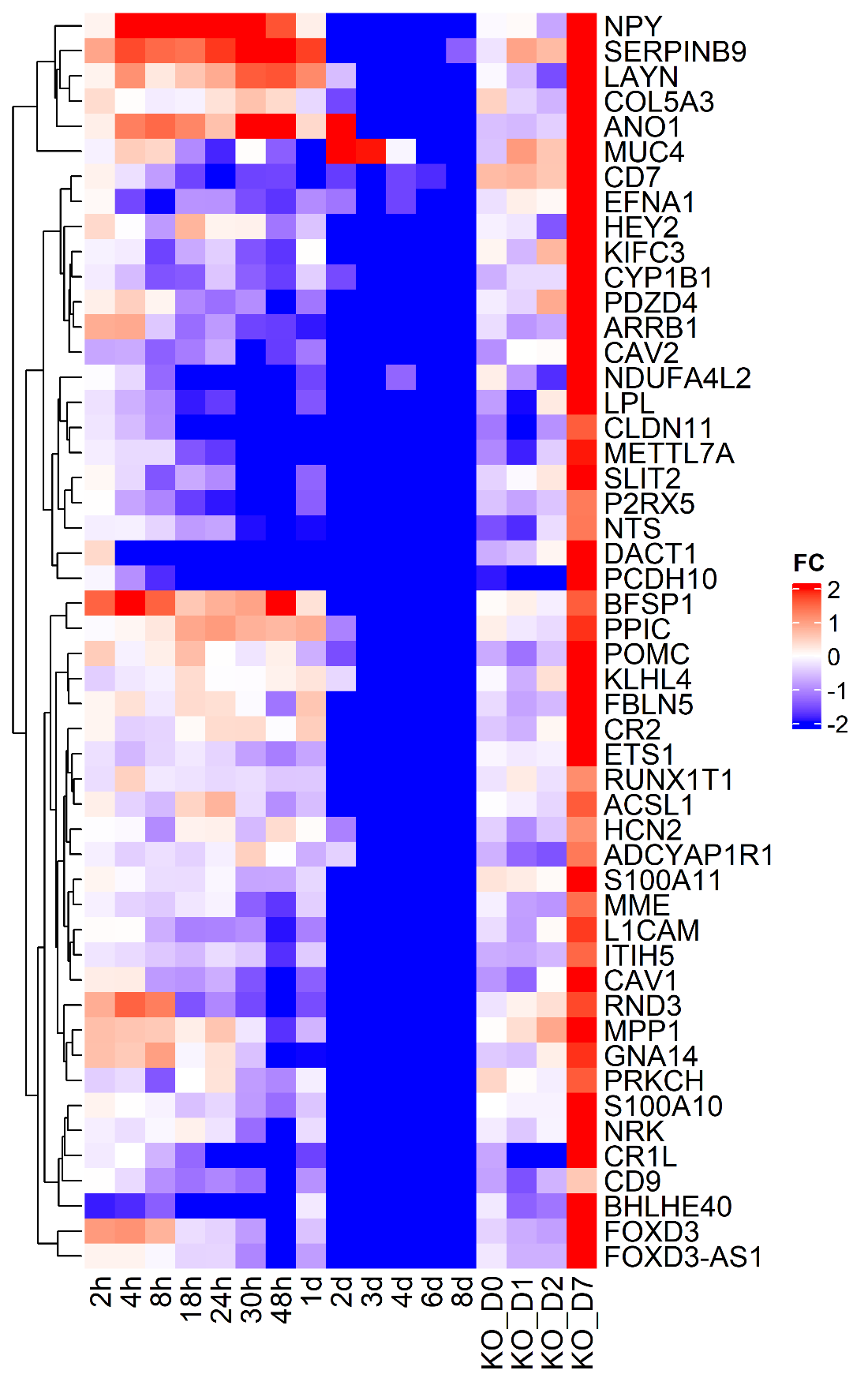


**Fig S6. Heatmap of the top 50 upregulated genes to show the effect of OTX2 KD.**

Table S1. Table of primers used for Q-PCR.

| **Gene** | **Primer Sense 3’-5’** | **Primer Antisense 5’-3’** |
| --- | --- | --- |
| FGF8 | CAG GTC CTG GCC AAC AAG | CTC CTC GGA CTC GAA CTC TG |
| GAPDH | AGC CTC AAG ATC ATC AGC AA | CTG TGG TCA TGA GTC CTT CC |
| HESX1 | TGC TTT TAC TCA AAA CCA GAT TGA | CCA AAT CTG GAT TCT GTC TTC C |
| NESTIN | AGG ACA CCA TGA GGA ACA GC | GCC ATG TTC TTG CTC ACG TC |
| NPTX1 | GTG ATA GGG CGC CAA GTT CT | ATC AAT GAC AAG GTG GCC AAG |
| OCT4 | TTG GGC TCG AGA AGG ATG TG | GTG AAG TGA GGG CTC CCA TA |
| PAX6 | GCC AGA GCC AGC ATG CAG AAC A | CCT GCA GAA TTC GGG AAA TGT CG |
| SIX3 | AGC AGA AGG ACC GAG TTC TG | CAA GAA CAG GCT CCA GCA C |
| SIX6 | GCT GCA GCC AAG AAC AGA CT | CTG GAC GTG ATG GAG ATG G |

Table S2. List of antibodies used for immunofluorescence.

| **Antibody** | **Type** | **Host** | **Dilution** | **Supplier** | **Catalog Number** |
| --- | --- | --- | --- | --- | --- |
| NESTIN | Monoclonal | Mouse | 1:500 | R&D Systems | MAB1259 |
| NR2F2 | Monoclonal | Mouse | 1:100 | R&D Systems | PP-H7147-00 |
| OCT4 | Monoclonal | Mouse | 1:200 | Santa Cruz | Sc5279 |
| PAX6 | Polyclonal | Rabbit | 1:200 | Proteintech | 12323-1-AP |
| ZEB1 | Monoclonal | Mouse | 1:300 | Atlas Antibodies | AMAb90510 |
| ZEB2 | Polyclonal | Rabbit | 1:150 | Atlas Antibodies | HPA003456 |
| ZNF521 | Polyclonal | Rabbit | 1:100 | Atlas Antibodies | HPA023056 |
